# Complete Genome Sequence of Acinetobacter baumannii Phage BS46

**DOI:** 10.1128/MRA.00398-20

**Published:** 2020-05-28

**Authors:** Anastasia V. Popova, Mikhail M. Shneider, Yulia V. Mikhailova, Andrey A. Shelenkov, Dmitry A. Shagin, Mikhail V. Edelstein, Roman S. Kozlov

**Affiliations:** a Moscow Institute of Physics and Technology (National Research University), Dolgoprudny, Moscow Region, Russia; b Institute of Antimicrobial Chemotherapy, Smolensk State Medical University, Smolensk, Russia; c State Research Center for Applied Microbiology and Biotechnology, Obolensk, Moscow Region, Russia; d Shemyakin-Ovchinnikov Institute of Bioorganic Chemistry, Moscow, Russia; e Central Scientific Research Institute of Epidemiology, Moscow, Russia; f Pirogov Russian National Research Medical University, Moscow, Russia; DOE Joint Genome Institute

## Abstract

Acinetobacter myovirus BS46 was isolated from sewage by J. S. Soothill in 1991. We have sequenced the genome of BS46 and found it to be almost unique. BS46 contains double-stranded DNA with a genome size of 94,068 bp and 176 predicted open reading frames. The gene encoding the tailspike that presumably possesses depolymerase activity toward the capsular polysaccharides of the bacterial host was identified.

## ANNOUNCEMENT

Acinetobacter baumannii is characterized by an intrinsic resistance to many antibiotics and a high propensity to acquire secondary resistance to almost all antibiotic classes and therefore is regarded by the World Health Organization as a “critical priority” pathogen requiring the development of new strategies for effective therapy (1).

Acinetobacter phage BS46 was isolated from sewage (Birmingham, England) by Soothill in 1991 (2) and deposited in the Félix d’Hérelle Reference Centre for Bacterial Viruses at Laval University (Québec, Canada), in 1993 under host index number (HER) 401. According to a study published in 1994, BS46 was assigned to the family *Myoviridae* based on phage morphology (3). The phage has been shown to be effective in murine models (2) and has demonstrated no cytotoxic effect on mouse fibroblast 3T3 cells (4). However, despite the fact that phage BS46 was isolated more than 25 years ago, no data about the nucleotide sequence and organization of its genome have been published. Therefore, we aimed to investigate the BS46 genome structure and compare its nucleotide sequence with those of other Acinetobacter phages isolated and described in recent years.

Phage BS46 and its bacterial host, A. baumannii AC54 (5), were received from the Félix d’Hérelle Reference Centre for Bacterial Viruses at Laval University (Québec, Canada). Phage DNA was isolated from concentrated and purified high-titer phage stocks by incubation in 0.5% SDS, 20 mM EDTA, and 50 μg of proteinase K per ml at 65°C for 1 h. The DNA was extracted with phenol-chloroform and then precipitated with ethanol (6). Genome sequencing was performed on the MiSeq platform using a Nextera DNA library preparation kit (Illumina, San Diego, CA). In total, 195,536 200-bp single-end reads were obtained. All reads were subjected to quality-trimming using FLEXBAR software v. 2.5 (7) and then assembled *de novo* into a single contig using SPAdes v. 3.13 (8) with default parameters. The average coverage of the final contig was 185×.

Phage BS46 has a 94,068-bp linear double-stranded DNA genome with a G+C content of 33.5%. A total of 176 open reading frames (ORFs) were predicted using RAST (9). The search for tRNA sequences using ARAGORN (10) revealed 3 tRNAs for Tyr, Leu, and Arg.

Predicted proteins were searched against the NCBI nonredundant (nr) database, and an HHpred profile-profile search was conducted (11). On the basis of homology to the amino acid sequences of known phage proteins, putative functions were assigned for the products of 57 predicted genes, including proteins involved in nucleotide metabolism, transcription regulation, DNA replication, packaging of DNA into the capsid, host lysis, phage assembly, and structural proteins. Four ORFs encoding putative HNH endonucleases were found throughout the phage genome. No genes encoding toxins or factors responsible for antibiotic resistance were identified.

BLAST (12) analysis revealed that the complete genome sequence of phage BS46 appeared to be rather unique, sharing a homologous region containing tail structural component genes only with the recently characterized A. baumannii myovirus vB_AbM_B9 (GenBank accession number MH133207) (13). However, the gene products predicted to determine the host specificity, tailspike proteins, or structural depolymerases, gp47 of phage BS46 and gp69 of phage vB_AbM_B9, were found to differ significantly, sharing sequence similarity only in the *N*‐terminal domains responsible for attachment to phage particles. At the same time, the closest homolog of the BS46 tailspike protein was found to be the tailspike protein of Acinetobacter myophage AM24 (AM24_gp50; GenBank accession number APD20249), which infects strains with a K9 capsular polysaccharides structure (14). This, most likely, indicates that the tailspike of phage BS46 can interact specifically with capsular polysaccharides of the same structure.

### Data availability.

The complete genome sequence of Acinetobacter phage BS46 has been deposited in GenBank under accession number MN276049, BioProject accession number PRJNA562545, SRA accession number SRP219502, and BioSample accession number SAMN12643920. The version described in this paper is the first version.
